# Brain reserve and physical disability in secondary progressive multiple sclerosis

**DOI:** 10.1136/bmjno-2024-000670

**Published:** 2024-09-07

**Authors:** Nevin John, Yingtong Li, Floriana De Angelis, Jonathan Stutters, Ferran Prados Carrasco, Arman Eshaghi, Anisha Doshi, Alberto Calvi, Thomas Williams, Domenico Plantone, Thanh Phan, Frederik Barkhof, Jeremy Chataway, for the MS-SMART Investigators, Sebastien Ourselin

**Affiliations:** 1 Department of Medicine, School of Clinical Sciences, Monash University, Clayton, Victoria, Australia; 2 Department of Neurology, Monash Health, Clayton, Victoria, Australia; 3 Queen Square Multiple Sclerosis Centre, Department of Neuroinflammation, UCL Institute of Neurology, Faculty of Brain Sciences, University College London, London, UK; 4 Translational Imaging Group, Centre for Medical Image Computing (CMIC), Department of Medical Physics and Bioengineering, University College London, London, UK; 5 e-Health Center, Universitat Oberta de Catalunya, Barcelona, Spain; 6 Advanced Imaging in Neuroimmunological Diseases Lab (ImaginEM), Fundacio Clinic per la Recerca Biomedica, Barcelona, Spain; 7 Department of Medicine, Surgery & Neuroscience, University of Siena, Siena, Italy; 8 NIHR University College London Hospitals Biomedical Research Centre, London, UK; 9 Department of Radiology and Nuclear Medicine, Amsterdam University Medical Centre, Amsterdam, The Netherlands

**Keywords:** MULTIPLE SCLEROSIS, NEUROIMMUNOLOGY

## Abstract

**Background:**

The brain reserve hypothesis posits that larger maximal lifetime brain growth (MLBG) may confer protection against physical disability in multiple sclerosis (MS). Larger MLBG as a proxy for brain reserve, has been associated with reduced progression of physical disability in patients with early MS; however, it is unknown whether this association remains once in the secondary progressive phase of MS (SPMS). Our aim was to assess whether larger MLBG is associated with decreased physical disability progression in SPMS.

**Methods:**

We conducted a post hoc analysis of participants in the MS-Secondary Progressive Multi-Arm Randomisation Trial (NCT01910259), a multicentre randomised placebo-controlled trial of the neuroprotective potential of three agents in SPMS. Physical disability was measured by Expanded Disability Status Scale (EDSS), 9-hole peg test (9HPT) and 25-foot timed walk test (T25FW) at baseline, 48 and 96 weeks. MLBG was estimated by baseline intracranial volume (ICV). Multivariable time-varying Cox regression models were used to investigate the association between MLBG and physical disability progression.

**Results:**

383 participants (mean age 54.5 years, 298 female) were followed up over 96 weeks. Median baseline EDSS was 6.0 (range 4.0–6.5). Adjusted for covariates, larger MLBG was associated with a reduced risk of EDSS progression (HR 0.84,95% CI:0.72 to 0.99;p=0.04). MLBG was not independently associated with time to progression as measured by 9HPT or T25FW.

**Conclusion:**

Larger MLBG is independently associated with physical disability progression over 96 weeks as measured by EDSS in SPMS. This suggests that MLBG as a proxy for brain reserve may continue to confer protection against disability when in the secondary progression phase of MS.

**Trail registration number:**

NCT01910259.

**WHAT IS ALREADY KNOWN ON THIS TOPIC**

- Increased brain reserve measured using maximal lifetime brain growth as a proxy has been shown to be associated with decreased physical disability progression in early relapsing remitting multiple sclerosis (MS). This study examines whether increased brain reserve may protect against physical disability progression once in the secondary progressive phase of MS.

**WHAT THIS STUDY ADDS**

- This study shows that increased brain reserve may confer protection against physical disability progression even once in the secondary progressive phase of MS. This has not been demonstrated before.

**HOW THIS STUDY MIGHT AFFECT RESEARCH, PRACTICE OR POLICY**

- Our study provides impetus for additional research exploring factors in brain reserve and studying the combined effects of brain, cognitive and functional reserve and their contribution to physical and cognitive disability progression in MS.

## Introduction

 The brain reserve hypothesis suggests that larger maximal lifetime brain growth (MLBG) may confer protection against cognitive impairment and physical disability in neurological conditions.^1 2^ MLBG is predominantly genetically determined and usually reaches its maximum by 10 years of age.^3 4^ The brain reserve hypothesis was initially studied in dementia and Parkinson’s disease before the concept of threshold factor and brain reserve was formalised into a theoretical construct by Satz to explain the interindividual variability in clinical symptoms between those with similar levels of brain pathology.^1^ He outlined the core concept of brain reserve capacity and described a number of hypotheses relating brain reserve capacity and its association with symptom onset in disease. These include greater brain reserve capacity being a protective factor; which people with reduced brain reserve may be more vulnerable to clinical symptoms with premorbid insults, further contributing to this vulnerability.^1^

Brain reserve has an anatomical proxy with initial studies in Alzheimer’s dementia, showing those with larger head circumference having a 20% decreased risk of developing the disease after adjusting for age, education, ethnicity, gender and height.^5 6^ Larger MLBG is associated with larger neuronal count, which may be more robust to disease-related disruption and provide additional plasticity to respond to disease.^7^ This concept was also supported by a postmortem study that described a group of subjects with pathological evidence of Alzheimer’s disease with minimal symptoms. This group had higher brain weight and greater number of neurons, leading to the hypothesis that larger brain size may be protective through ‘greater reserve’.^8^ The introduction of MRI then enabled the use of total intracranial volume as a proxy for brain reserve or MLBG with this being the most widely used method particularly in dementia studies.^1 3 9^

In multiple sclerosis (MS), the concept of brain reserve was initially studied in a cross-sectional study of 62 people demonstrating that smaller intracranial volume was associated with slower cognitive processing speed.^10^ The follow-up to this initial study (n=40) showed that greater brain reserve protected against deterioration in cognitive processing speed.^11^ The only other longitudinal study in MS demonstrated that greater brain reserve was associated with decreased progression in physical disability in people with early MS.^2^ However, it is unknown whether association between brain reserve and physical disability remains once in the secondary progressive phase of the disease (SPMS).

The Multiple Sclerosis—Secondary Progressive Multi-Arm Trial (MS-SMART) was a phase 2b multiarm, multicentre, randomised placebo-controlled trial evaluating the neuroprotective potential of amiloride, fluoxetine and riluzole in SPMS (NCT01910259). Results of this trial have been published previously, with none of the three treatment arms demonstrating therapeutic effect on the primary endpoint of percentage brain volume change (PBVC) over 96 weeks (atrophy).^12^ We now use this cohort to examine the association between MLBG and physical disability progression. We hypothesise that larger brain reserve at baseline will be associated with decreased physical disability progression.

## Methods

### Study participants

We conducted a post hoc analysis of participants from the MS-SMART trial. The inclusion/exclusion criteria and primary/secondary outcome measures have been previously described.^13^ In brief, eligible participants were aged 25–65 with an Expanded Disability Status Scale (EDSS) score of 4.0–6.5, who showed evidence of progression of SPMS over the preceding 2 years, which was independent of relapses. Participants were recruited from 13 UK neuroscience centres between 29 January 2015 and 22 June 2016. Participants were randomised to amiloride, fluoxetine, riluzole or placebo; and the primary outcome measure was per cent PBVC over 96 weeks.^12 13^ The sample size calculation for the MS-SMART trial has been outlined previously.^13^

### Physical disability variables

Physical disability was measured by EDSS, 9-hole peg test (9HPT) and timed 25-foot timed walk (T25FW) at baseline, 48 and 96 weeks by assessors blinded to treatment allocation. Clinically significant progression was defined as increase in EDSS score of ≥1.0 if baseline ≤5.0 or ≥0.5 if baseline ≥5.5, or increase in 9HPT or T25FW time of≥20%.

### MRI acquisition and analysis

Participants were scanned on 3Tesla MRI scanners using a standardised MRI acquisition protocol. The acquisition protocol included PD/T2, fluid attenuated inversion recovery (FLAIR) and T1-weighted sequences and has been described previously.^13^ T2 lesion volume (T2LV) was measured using Jim7 (Xinapse, UK) software, after lesion filling.^14^ MLBG estimated by baseline intracranial volume (ICV) used as a proxy of brain reserve was obtained using SIENAX (Structural Image Evaluation, using Normalisation, of Atrophy X) and includes the brain, meninges and cerebrospinal fluid (CSF).^15^ Specifically, brain and skull images were extracted from single whole-head input data, and tissue-type segmentation with partial volume estimation was performed to yield a volumetric measure of ICV.^16^ ICV was then standardised as *z* score, using the mean and SD for men and women, respectively. ICV was additionally classified as larger or smaller based on median ICV for sex to be concordant with previous studies.^2^ PBVC over 96 weeks was measured using SIENA.^15^ Normalised brain volume (NBV) was also measured using the SIENAX method.

### Statistical analysis

In a complete-case analysis, we first performed univariable analyses comparing the characteristics of participants who did and did not experience progression in EDSS over 96 weeks. Continuous variables, namely age, MLBG, baseline NBV, and PBVC over 96 weeks, were compared using Student’s t test where approximate normality could be assumed. Baseline EDSS and T2LV could not be assumed to be normal and were compared using the Mann-Whitney U test. Categorical variables, namely sex, relapse within the preceding 2 years, randomised treatment group and the presence of new/enlarging T2 lesions over 96 weeks, were compared using the Pearson χ^2^ test.

We then used multivariable time-varying Cox regression models to investigate the association between MLBG and time to physical disability progression in EDSS, 9HPT and T25FW; adjusting for age, sex, baseline NBV, baseline T2LV, PBVC, relapse within 2 years and any other covariates significant in the univariable analysis. The brain reserve concept aims to explain the difference between the observed and expected physical performance based on the underlying level of pathology. Therefore, models should include a measure of neuropathology for which NBV and T2LV were included as covariates.^9^ Results of the regression are presented as hazard ratios (HR). Study trial allocation was not included as a covariate as there was no effect of any of the three agents on the primary outcome of percentage PBVC.^12^

In secondary analyses, we used Cox regression models to investigate the association between categorisation of MLBG as larger or small and time to physical disability progression in EDSS, adjusting for the same covariates. We also evaluate the relative contribution of MLBG and each covariate in the Cox model to the risk of disability progression by calculating Shapley Additive exPlanations (SHAP) values. Analysis using SHAP values is based on cooperative game theory.^17^ The marginal contribution or Shapley value is determined as the average of all permutations of the coalition of the covariates containing the covariate of interest minus the coalition without the covariate of interest.^18^ In the context of regression, it assigns an importance value to each regressor, which represents the additive marginal effect on the model prediction of including that regressor. The network relationships among the variables were examined by building a partial correlation network. Multivariable linear regression was used to investigate the association between MLBG and change in EDSS score over 96 weeks, adjusting for the same covariates. Results of the regression are presented as adjusted mean differences.

The statistical analyses were performed using Python (SciPy V.1.10.1, statsmodels 0.13.5 and lifelines 0.27.7). All significance tests were two-sided, and p values <0.05 were considered significant.

## Results

### Participants

445 participants were enrolled in the trial. Mean age was 54.5 years (SD 7.0) and 298/445 (67.0%) were women. Median baseline EDSS was 6.0 (IQR 6.0–6.5). 383 of 445 enrolled participants (86.1%) were followed up over 96 weeks. 155/445 (35%) had previously used any disease-modifying therapy with 142/445 (32%) using first-line treatment and 26/445 (6%) using second-line therapy. Table 1 summarises the baseline characteristics of the enrolled participants.

**Table 1:** Baseline characteristics of participants

Age in years, mean (SD)	54.5 (7.0)
Female, n (%)	298 (67%)
EDSS, median (IQR)	6.0 (6.0–6.5)
ICV (MLBG), cm^3^, mean (SD)	1513.6 (137.7)
ICV (MLBG), large:small	223:222
Baseline NBV, cm^3^, mean (SD)	1423.6 (83.6)
Baseline T2LV, cm^3^, median (IQR)	10.4 (4.1–18.6)
Relapse in preceding year (n, %)	43 (10%)

None of the participants commenced disease modifying therapy (aside of the study agents) during the trial.

EDSS, Expanded Disability Status Scale; ICV, intracranial volume; MLBG, maximal lifetime brain growth; NBV, normalised brain volume; T2LV, T2 lesion volume.

62 enrolled participants did not undergo 96-week EDSS assessment due to formal withdrawal (16/62), loss to follow-up (43/62) and death unrelated to the study agents (3/62). The baseline characteristics of those with missing follow-up at 96 weeks were similar to those with complete follow-up.

### EDSS progression

Over 96 weeks, 150/383 participants (39.2%) demonstrated clinically significant progression in EDSS.

In univariable analysis, younger age (t(381)=2.19; p=0.03), male sex (χ^2^(1)=7.43; p=0.006), lower baseline EDSS (Mann-Whitney p<0.001) and smaller MLBG (χ^2^(1)=2.10; p=0.04) were associated with an increased risk of EDSS progression (online supplemental table 1).

Over 96 weeks, 81/383 progressed on 9HPT, and 169/383 progressed on T25FW.

### Multivariable analyses

In the Cox regression model, larger MLBG was independently associated with reduced hazard of EDSS progression (HR 0.84, per SD increase in MLBG, 95% CI 0.72 to 0.99; p=0.04, table 2).

**Table 2:** Factors associated with time to EDSS progression, continuous MLBG, multivariable regression

	HR (95% CI)	P
Larger MLBG, per SD increase	0.84 (0.72 to 0.99)	0.04*
Male sex	1.60 (1.12 to 2.27)	0.009*

Multivariable Cox regression. Likelihood ratio test p=0.02.

Non-significant covariates omitted from the table: age, baseline NBV, baseline T2LV, relapse within previous 2 years, PBVC.

* p<0.05.

EDSS, expanded disability status scale; MLBG, maximal lifetime brain growth; PBVC, percentage brain volume change; T2LV, T2 lesion volume.

**Figure 1:**
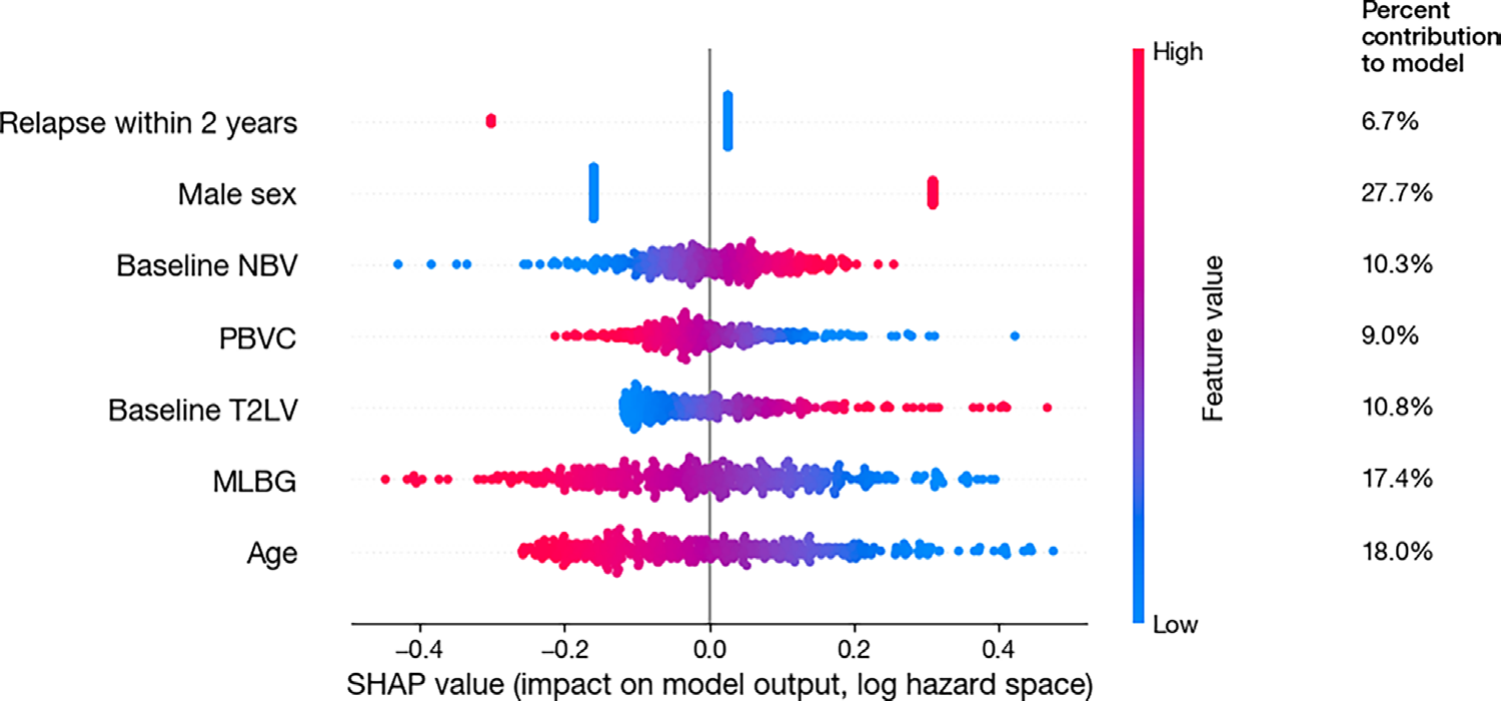
SHAP analysis of factors associated with risk of EDSS progression SHAP values for parameters in the Cox regression evaluating the association between MLBG and EDSS progression. The column on the right shows the mean absolute SHAP value for each parameter, as a percentage of the total, representing the percent contribution of each parameter to the model output. The x-axis shows the SHAP values with each individual dot signifying SHAP value of a particular feature for a given data point. For the purposes of SHAP illustration, age is presented as a continuous covariate and PBVC is presented as at 96 weeks. EDSS, expanded disability status scale; MLBG, maximal lifetime brain growth; NBV, normalised brain volume; PBVC, percent brain volume change; SHAP, SHapley Additive exPlanations; T2LV, T2 lesion volume.

**Figure 2:**
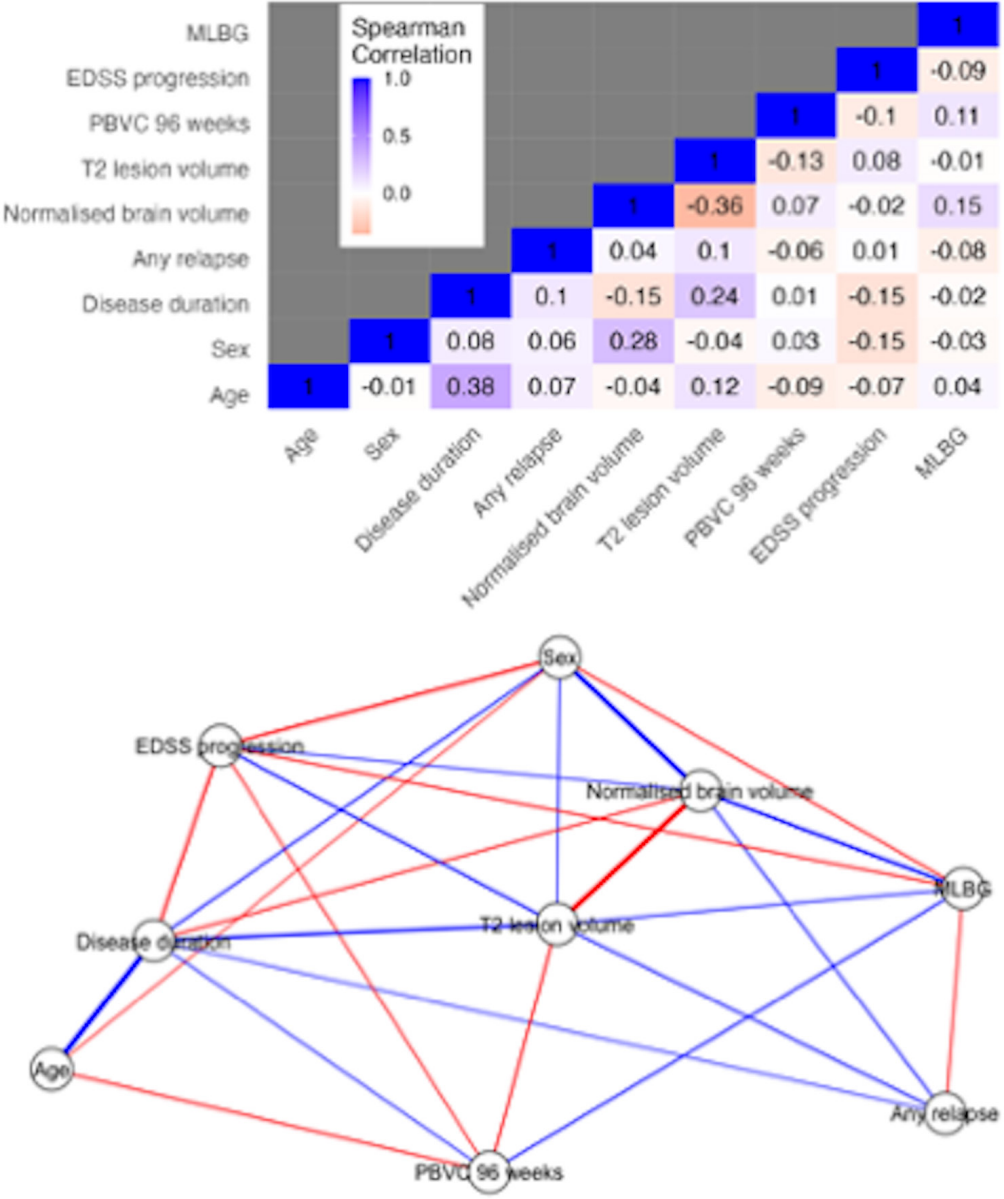
Correlation matrix and correlation network of EDSS progression. The top row is the correlation matrix representing the correlation coefficients between the variables. Positive correlations are blue and negative correlations shown as red. The bottom row is the correlation network and shows the interactions between the variables. Again, positive correlations are labelled in blue and negative correlations are in red. The thickness of the line represents the strength of the partial correlation. EDSS, expanded disability status scale; MLBG, maximal lifetime brain growth; NBV, normalised brain volume; PBVC, percent brain volume change.

The SHAP values show that MLBG made the second largest contribution to increasing the hazard of EDSS progression, contributing 17.4% to the model output; with male sex contributing 27.7% (figure 1). The figure also demonstrates the relationship of each variable and the model output—hazard of EDSS progression. Figure 1 demonstrates that larger MLBG predicts a decreased hazard of EDSS progression, whereas smaller MLBG predicts an increased hazard; and being male predicts an increased hazard of progression compared with women (figure 1). The correlation matrix and the partial correlation network highlighting the relationship among the variables are shown in figure 2. Age was moderately correlated to disease duration (σ=0.38), which in turn showed a low negative correlation with EDSS progression (σ=−0.15). Both MLBG (σ=−0.09) and PBVC over 96 weeks (σ=−0.10) showed negative correlations with EDSS progression.

In the secondary analysis, larger MLBG than median was independently associated with reduced hazard of EDSS progression (HR 0.62, 95% CI 0.45 to 0.87; p=0.005, table 3).

**Table 3:** Factors associated with time to EDSS progression, dichotomous MLBG, multivariable regression

	HR (95% CI)	P
Larger MLBG than median	0.62 (0.45 to 0.87)	0.005*
Male sex	1.59 (1.12 to 2.25)	0.01*

Multivariable Cox regression. Likelihood ratio test p=0.006.

Nonsignificant covariates omitted from the table: age, baseline NBV, baseline T2LV, relapse within previous 2 years, PBVC.

* p<0.05.

EDSS, expanded disability status scale; MLBG, maximal lifetime brain growth; PBVC, percentage brain volume change.

Participants with larger MLBG than median also had smaller changes in EDSS score over 96 weeks on average (adjusted mean difference 0.16, 95% CI 0.01 to 0.31; p=0.04, table 4).

**Table 4:** Factors associated with 96-week change in EDSS scores, multivariable regression

	aMD (95% CI)	P
Larger MLBG than median	−0.16 (−0.31 to −0.01)	0.04*
Male sex	0.18 (0.02 to 0.35)	0.03*
PBVC, per percentage point volume increase	−0.07 (−0.13 to −0.02)	0.01*

Multivariable linear regression. R2=0.06. Likelihood ratio test p=0.007.

Non-significant covariates omitted from the table: age, baseline NBV, baseline T2LV, relapse within previous 2 years.

* p<0.05.

aMD, adjusted mean difference; EDSS, expanded disability status scale; MLBG, maximal lifetime brain growth; PBVC, percent brain volume change.

No association was seen between MLBG and time to progression as measured by 9HPT (HR 1.00, per SD, 95% CI 0.80 to 1.25; p=0.98) or T25FW (HR 1.03, 95% CI 0.88 to 1.20; p=0.75).

## Discussion

Extending the brain reserve hypothesis to physical disability in a large randomised control trial cohort with SPMS, we demonstrate that patients with larger MLBG were at lower risk of physical disability progression over 96 weeks. This suggests that MLBG as a proxy for brain reserve continues to confer protection against physical disability in SPMS. Consideration of MLBG may also help stratify participants at greater risk of disability progression in clinical trials of neuroprotective agents in progressive forms of MS, thereby facilitating a more efficient trial design.

We used SHAP analysis often used in machine learning to further interpret the individual contribution of each variable in predicting EDSS progression, which extends our understanding of the results gained from Cox regression. This novel approach to the research question enabled an improved understanding of MLBG and the other covariates by showing that being female and having larger MLBG were the most important features when predicting EDSS progression in SPMS. We then extended this by using a network approach to understand the relationship between the key variables in the model and our dependent variable (EDSS progression). We did this by creating a partial correlation network. Figure 2b illustrates the expected negative association between MLBG and EDSS progression. It also shows that age was positively correlated with disease duration, which in turn was negatively associated with EDSS progression. Baseline NBV was negatively associated with T2LV, while disease duration was positively associated with T2LV then showing a positive association with EDSS progression.

Our findings build on the previous work by Sumowski *et al* by demonstrating for the first time that patients with larger MLBG had a lower risk of physical disability progression in adults with SPMS.^2^ Sumowski *et al* showed that larger MLBG was associated with a lower risk of physical disability progression (measured using EDSS) over 5 years in adults with MS. Compared with our study, their cohort was younger (mean 43 years), had a shorter disease duration (mean 10.3 years), lower level of disability (median EDSS 3.5) and contained predominantly RRMS (32 RRMS out of 52).^2^ Our study duration was comparatively shorter, yet we were able to demonstrate that larger MLBG was associated with a decreased risk of disability progression over this shorter time frame.

MLBG is not thought to increase after 10 years and is thought to be genetically determined therefore representing a static or fixed concept.^4^ Previous longitudinal studies demonstrated that grey matter volumes peak at 4 years while cortical white matter volumes peak by 20 years of age.^4 9^ There is debate about whether neurogenesis can occur in adulthood but there is evidence of neurogenesis and plasticity in specific areas such as the hippocampus and subventricular regions.^19^ This may be influenced by factors such as exercise, diet and stressors.^9^ There is also some evidence that early and perinatal environmental factors may have an additional role in determining MLBG.^20^ These include malnutrition and vitamin deficiencies in the early years of life,^21^ intrapartum factors such as maternal stress,^22^ and exposures to potential toxins (alcohol) and medication (eg, antiepileptics).^23^ This brings into consideration the concept of brain reserve and whether it is static where MLBG does not change or whether it is dynamic and influenced by certain environmental factors. There is also a separate but possibly interlinked hypothesis of functional brain reserve whereby those with a greater potential for neuroplasticity may be more resilient to the effects of neuropathology such as MS. Furthermore, it is of interest whether interventions such as regular exercise or physiotherapy promote neuroplasticity or whether we can interrogate brain reserve and neuroplasticity potential to determine who may benefit most from MS treatment strategies, particular rehabilitation; and physical therapies. Further study is needed to improve our understanding of these concepts and whether optimising comorbidities such as maternal depression and nutrition in the perinatal period that impact early brain development can influence brain reserve.^24 25^

The mechanisms through which greater brain reserve may be associated with decreased progression in MS and other neurodegenerative disease remains incompletely understood. There are several postulated models: First, the ‘threshold concept’ whereby brain reserve provides a higher threshold before the clinical effects of the underlying pathology become evident.^1^ Second, the initial advantage model whereby those with greater brain reserve must undergo greater levels of decline before objective clinical impairment is seen. Third, the concept of brain resilience reflecting the ability of the brain to cope with accumulating pathology and maintain physical and/or cognitive performance.^26^ MS-specific hypotheses include the topographical model, whereby clinical symptoms occur once a functional reserve is crossed and that the progression is encapsulated by focal inflammatory activity in eloquent regions of the brain and spine.^27^ Vollmer *et al* later outlined neurological reserve in MS, whereby clinical disease progression is unmasked once subclinical inflammatory activity and biological ageing overcome brain and cognitive reserve.^28^ It is also of interest to examine the link between (active) cognitive reserve, brain reserve and cognitive performance in MS as previous studies in dementia have demonstrated that those with cognitive reserve have greater positive effects in those with greater brain reserve (measured using ICV).^9^

The strengths of the study include a well-phenotyped cohort typical of SPMS who were recruited into a randomised clinical trial that underwent rigorous assessment using both assessments of disability and MRI measures over 96 weeks. Participants were not on disease modifying therapy at study entry, they did not commence disease-modifying therapy during the trial, and none of the treatment arms decreased whole brain atrophy or physical disability progression. Therefore, the study could be considered a natural history study of SPMS. We also incorporated additional measures of upper limb function (9HPT) and walking speed (T25FW) that have not been investigated previously. There are several limitations—no association was found between larger brain reserve and upper limb function likely due to the small proportion of participants that demonstrated a worsening in 9HPT times over 96 weeks. There was also no association between progression on T25FW and larger brain reserve, which may be due to differences in the outcome measures where the 9HPT, T25FW and EDSS may not always be concordant.^29^ An example of this is seen in the EXPAND trial in SPMS where siponimod decreased EDSS-confirmed disability progression but had no effect on T25FW progression rates^30^; a similar result was seen in the phase II MS-STAT trial of high-dose simvastatin in SPMS.^31^ Conversely, the discordance represents the possibility that our association is due to chance. This was a post hoc analysis and was, therefore, not powered specifically for this study. The study followed participants for 96 weeks, which may not capture long-term disability progression in SPMS, limiting our ability to assess the sustained impact of brain reserve on disability over longer time periods. It would also be of interest to incorporate functional reserve, which may be measured using functional connectivity, environmental factors such as socioeconomic measures and stressful early life exposures to better understand their association with physical disability alongside MLBG.^28^ There are considerations about the use of MLBG measured using ICV as a proxy for brain reserve. For example, total ICV may not capture subtle individual variation in cortical surface area; and automated measures using the most commonly used software (FSL, Freesurfer and SPM12) have shown excellent reliability and accuracy compared with manual delineation methods but may still be confounded by factors such as sex.^9 32 33^

In summary, we extend the brain reserve hypothesis to physical disability progression in adults with SPMS, demonstrating that greater brain reserve may provide a protective role against physical disability progression once in SPMS.

## Supplementary material

10.1136/bmjno-2024-000670 online supplemental table 1

## Data Availability

Data are available upon reasonable request.

## References

[1] Satz P (1993). Brain reserve capacity on symptom onset after brain injury: a formulation and review of evidence for threshold theory. Neuropsychology.

[2] Sumowski JF, Rocca MA, Leavitt VM (2016). Brain reserve against physical disability progression over 5 years in multiple sclerosis. Neurol (ECronicon).

[3] de Rooij SR (2022). Are Brain and Cognitive Reserve Shaped by Early Life Circumstances?. Front Neurosci.

[4] Pfefferbaum A, Mathalon DH, Sullivan EV (1994). A quantitative magnetic resonance imaging study of changes in brain morphology from infancy to late adulthood. Arch Neurol.

[5] Schofield PW, Logroscino G, Andrews HF (1997). An association between head circumference and Alzheimer’s disease in a population-based study of aging and dementia. Neurology (ECronicon).

[6] Stern Y (2002). What is cognitive reserve? Theory and research application of the reserve concept. J Int Neuropsychol Soc.

[7] Haug H (1987). Brain sizes, surfaces, and neuronal sizes of the cortex cerebri: a stereological investigation of man and his variability and a comparison with some mammals (primates, whales, marsupials, insectivores, and one elephant). Am J Anat.

[8] Katzman R, Terry R, DeTeresa R (1988). Clinical, pathological, and neurochemical changes in dementia: a subgroup with preserved mental status and numerous neocortical plaques. Ann Neurol.

[9] van Loenhoud AC, Groot C, Vogel JW (2018). Is intracranial volume a suitable proxy for brain reserve?. Alzheimers Res Ther.

[10] Sumowski JF, Rocca MA, Leavitt VM (2013). Brain reserve and cognitive reserve in multiple sclerosis: what you’ve got and how you use it. Neurol (ECronicon).

[11] Sumowski JF, Rocca MA, Leavitt VM (2014). Brain reserve and cognitive reserve protect against cognitive decline over 4.5 years in MS. Neurol (ECronicon).

[12] Chataway J, De Angelis F, Connick P (2020). Efficacy of three neuroprotective drugs in secondary progressive multiple sclerosis (MS-SMART): a phase 2b, multiarm, double-blind, randomised placebo-controlled trial. Lancet Neurol.

[13] Connick P, De Angelis F, Parker RA (2018). Multiple Sclerosis-Secondary Progressive Multi-Arm Randomisation Trial (MS-SMART): a multiarm phase IIb randomised, double-blind, placebo-controlled clinical trial comparing the efficacy of three neuroprotective drugs in secondary progressive multiple sclerosis. BMJ Open.

[14] Prados F, Cardoso MJ, Kanber B (2016). A multi-time-point modality-agnostic patch-based method for lesion filling in multiple sclerosis. Neuroimage.

[15] Smith SM, Zhang Y, Jenkinson M (2002). Accurate, robust, and automated longitudinal and cross-sectional brain change analysis. Neuroimage.

[16] Zhang Y, Brady M, Smith S (2001). Segmentation of brain MR images through a hidden Markov random field model and the expectation-maximization algorithm. IEEE Trans Med Imaging.

[17] Gale D, Shapley LS (1962). College admissions and the stability of marriage. Am Math Mon.

[18] Lundberg SM, Allen PG, Lee S-I (2017). A unified approach to interpreting model predictions.

[19] Boldrini M, Fulmore CA, Tartt AN (2018). Human Hippocampal Neurogenesis Persists throughout Aging. Cell Stem Cell.

[20] Adams HHH, Hibar DP, Chouraki V (2016). Novel genetic loci underlying human intracranial volume identified through genome-wide association. Nat Neurosci.

[21] Cohen Kadosh K, Muhardi L, Parikh P (2021). Nutritional Support of Neurodevelopment and Cognitive Function in Infants and Young Children-An Update and Novel Insights. Nutrients.

[22] Engel SM, Berkowitz GS, Wolff MS (2005). Psychological trauma associated with the World Trade Center attacks and its effect on pregnancy outcome. Paediatr Perinat Epidemiol.

[23] Roussotte FF, Sulik KK, Mattson SN (2012). Regional brain volume reductions relate to facial dysmorphology and neurocognitive function in fetal alcohol spectrum disorders. Hum Brain Mapp.

[24] Zou R, Tiemeier H, van der Ende J (2019). Exposure to Maternal Depressive Symptoms in Fetal Life or Childhood and Offspring Brain Development: A Population-Based Imaging Study. Am J Psychiatry.

[25] Zou R, El Marroun H, Cecil C (2021). Maternal folate levels during pregnancy and offspring brain development in late childhood. Clin Nutr.

[26] Bocancea DI, van Loenhoud AC, Groot C (2021). Measuring Resilience and Resistance in Aging and Alzheimer Disease Using Residual Methods: A Systematic Review and Meta-analysis. Neurology (ECronicon).

[27] Krieger SC, Cook K, De Nino S (2016). The topographical model of multiple sclerosis: A dynamic visualization of disease course. Neurol Neuroimmunol Neuroinflamm.

[28] Vollmer TL, Nair KV, Williams IM (2021). Multiple Sclerosis Phenotypes as a Continuum: The Role of Neurologic Reserve. Neurol Clin Pract.

[29] Koch MW, Mostert J, Repovic P (2021). Reliability of Outcome Measures in Clinical Trials in Secondary Progressive Multiple Sclerosis. Neurology (ECronicon).

[30] Kappos L, Bar-Or A, Cree BAC (2018). Siponimod versus placebo in secondary progressive multiple sclerosis (EXPAND): a double-blind, randomised, phase 3 study. Lancet.

[31] Chataway J, Schuerer N, Alsanousi A (2014). Effect of high-dose simvastatin on brain atrophy and disability in secondary progressive multiple sclerosis (MS-STAT): a randomised, placebo-controlled, phase 2 trial. Lancet.

[32] Malone IB, Leung KK, Clegg S (2015). Accurate automatic estimation of total intracranial volume: a nuisance variable with less nuisance. Neuroimage.

[33] Guo C, Ferreira D, Fink K (2019). Repeatability and reproducibility of FreeSurfer, FSL-SIENAX and SPM brain volumetric measurements and the effect of lesion filling in multiple sclerosis. Eur Radiol.

