# Supplementary material for: Brain reserve and physical disability in secondary progressive multiple sclerosis
Source: BMJ Neurol Open. 2024 Sep 7;6(2):e000670. doi: 10.1136/bmjno-2024-000670 (PMC11387515; doi:10.1136/bmjno-2024-000670)
Supplement: online supplemental table 1 [file bmjno-6-2-s001.pdf]

Supplementary Table 1. Characteristics of participants with and without EDSS progression

	**No progression (n = 233)**	**Progression (n = 150)**	t / U / χ^2	p
Age in years, mean (SD)	55.4 (6.9)	53.8 (7.0)	$t(381) = 2.19$	0.03*
Female, n (%)	168 (68%)	88 (65%)	$\chi^2(1) = 7.43$	0.006*
Baseline EDSS, median (IQR)	6.0 (6.0–6.5)	6.0 (5.5–6.5)	$U = 13755$	<0.001*
MLBG, z score, mean (SD)	0.07 (0.98)	−0.15 (1.04)	$t(381) = 2.10$	0.04*
Baseline NBV, cm^3, mean (SD)	1422.6 (83.2)	1417.5 (83.9)	$t(381) = 0.47$	0.64
Baseline T2LV, cm^3, median (IQR)	13.2 (5.5–23.7)	15.4 (5.9–25.1)	$U = 16507$	0.36
Relapse in preceding year, yes:no	25:208	10:140	$\chi^2(1) = 1.81$	0.18
PBVC over 96 weeks, mean (SD)	−1.3 (1.3)	−1.5 (1.3)	$t(370) = 1.57$	0.12
New/enlarging T2 lesions over 96 weeks, yes:no	98:132	65:84	$\chi^2(1) = 0.04$	0.85

EDSS: Expanded Disability Status Scale. IQR: interquartile range. MLBG: maximal lifetime brain growth. NBV: normalized brain volume. PBVC: percent brain volume change. SD: standard deviation. T2LV: T2 lesion volume.

t: Student's *t* test. *U*: Mann–Whitney *U* test. χ^2: Pearson χ^2 test. *$p < 0.05$.
